# Oxygen Reduction Reaction Activity and Stability of Shaped Metal-Doped PtNi Electrocatalysts Evaluated in Gas Diffusion Electrode Half-Cells

**DOI:** 10.1021/acsami.4c11068

**Published:** 2024-09-19

**Authors:** Shlomi Polani, Raffaele Amitrano, Adrian Felix Baumunk, Lujin Pan, Jiasheng Lu, Nicolai Schmitt, Ulrich Gernert, Malte Klingenhof, Sören Selve, Christian M. Günther, Bastian J. M. Etzold, Peter Strasser

**Affiliations:** † Electrochemical Energy, Catalysis and Material Science Laboratory, Department of Chemistry, Technical University Berlin, Berlin 10623, Germany; ‡ Friedrich-Alexander-Universität Erlangen Nürnberg, Power-to-X Technologies, Dr.-Mack-Straße 81, Fürth 90762, Germany; § Center for Electron Microscopy (ZELMI), Technical University of Berlin, Berlin 10623, Germany; ∥ Ernst-Berl-Institute for Technical Chemistry and Macromolecular Science, Technical University of Darmstadt, Peter-Grünberg-Strasse 8, Darmstadt 64287, Germany

**Keywords:** octahedral PtNi/C electrocatalyst, metal doping, proton exchange membrane fuel cell, high performance and durability, rotating disk electrode, gas diffusion electrode, half-cell setup

## Abstract

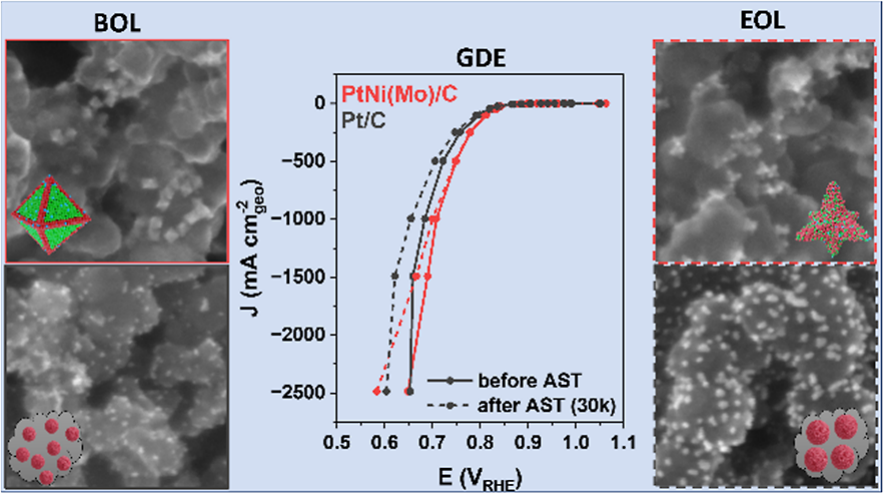

The synthesis of bimetallic and trimetallic platinum-based octahedral catalysts for the cathode of proton exchange membrane fuel cells (PEMFCs) is a particularly active area aimed at meeting technological requirements in terms of durability and cost. The electrocatalytic activity and stability of these shaped catalysts were tested at relatively high potentials (@0.9 V vs RHE) and at lower current densities using the rotating disk electrode, which is less suitable for assessing their behavior under the operating conditions of PEMFCs. In this work, we use a gas diffusion electrode (GDE) half-cell setup to test the performance of the catalysts under application-oriented conditions, relatively higher current densities, and a square-wave stability test. After the stability test, we analyzed the GDE catalytic layer to study the agglomeration and dissolution of the transition metal under these conditions by using high-resolution scanning electron microscopy and energy-dispersive X-ray spectroscopy. The present results provide valuable guidance for developing next-generation active and durable catalysts for PEMFCs.

## Introduction

Passenger cars powered by hydrogen fuel cells have reached the early stages of commercialization. Despite significant advantages in terms of refueling convenience and driving range, durability and cost remain the biggest challenges to broader fuel cell vehicle deployment. This is due to the high Pt loading at the fuel cell cathode required to compensate for the kinetically sluggish oxygen reduction reaction (ORR) (still about 0.3 mg_Pt_ cm^–2^).^1^ Therefore, current research is focused on identifying active and stable catalysts that can achieve and maintain the same or higher activity with lower Pt loading, preferably less than 0.125 mg_Pt_ cm^–2^ and 5000 h system durability with less than 10% loss of performance, the 2025 target of the United States Department of Energy (DOE).^2,3^

Among pure metals, Pt is the ideal catalyst for oxygen reduction. Alloying Pt with a transition metal not only lowers the cost of Pt-based catalysts but also greatly increases their catalytic activity due to geometric strain and electronic ligand effects.^4,5^ As a result of this, the electrocatalytic ORR reactivity of the platinum–nickel (111) single crystal facets^6^ is much superior to that of its (100) and (110) counterparts, ranging among the highest catalytic ORR activities ever recorded and reported.

The PtNi single-crystal work led to heightened interest in faceted PtNi nanocatalysts, particularly octahedral (oh-) catalysts with exclusive (111) orientation.^7^ Although these catalysts have shown ever increasing Pt mass activity using the liquid-electrolyte electrochemical rotating disk electrode (RDE) screening method, they suffer from poor stability. This is due to the acidic environment (pH < 1) and the very positive electrode potentials (>0.7 V vs RHE), which lead to selective metal corrosion and leaching of the transition metals and make the overall activity of the catalyst gradually approach that of pure Pt.^8^ In a membrane electrode assembly (MEA), relative humidity (RH) and the operating temperature have additional impacts on the dissolution of the non-noble metal and Pt mitigation. MEA tests with low RH have shown enhanced stability of a Pt alloy catalyst, whereas increasing operating temperatures led to growing degradation effects.^9,10^ To increase the stability of oh-PtNi catalysts, doping with a third metal has been shown to be effective.

While the RDE setup has been convenient and useful to reject catalyst candidates based on their low kinetic catalytic reactivity,^11^ this kinetic evaluation is limited to electrode potentials at low current densities commensurate with the oxygen solubility in the aqueous electrolyte. This is why the intrinsic activity of ORR catalysts can only be measured in a narrow potential range (0.85–0.95 V vs RHE) and at lower current densities compared to the operating conditions of a single MEA cell, the basic repeating unit in a fuel cell.^1^ Another shortcoming is the successful prediction of catalytic activity and materials transfer from the RDE level to single-cell MEA. Typically, for Pt alloys, such attempts have resulted in a more than 3-fold drop-in activity in MEAs, which has prevented a successful transfer of high RDE-performing, shape-controlled octahedral catalysts to a MEA.^12,13^ In addition, compared to the RDE, the fabrication of MEA requires a significant amount of catalyst for ink preparation and the deposition of the catalytic layer, so lab-scale synthesis must be scalable, which is not always straightforward, especially for shaped catalysts.^14^

Over the past years, the gas diffusion electrode (GDE) half-cell setup was introduced as a bridging technology between the RDE level and the MEA setup.^15^ Similar to the RDE, the GDE half-cell requires less than 5 mg of catalysts for ink preparation and can serve as a fast method for the electrochemical screening of catalysts.^16^ Furthermore, in a GDE flow cell, oxygen flows through one side of the electrode, while the other side is in a concentrated electrolyte. In this way, we can test the intrinsic activity of catalysts in a broader potential range (0.6–0.95 V vs RHE) and higher current densities (∼3000 mA cm^–2^) than the RDE, which are more relevant to the operation conditions of a proton exchange membrane fuel cell (PEMFC) cathode (0.65–0.8 V vs RHE), but still in a liquid medium.^15,17^ In a recent study, it was shown that the GDE half-cell setup is able to correctly predict trends for the MEA activity, which underlines its potential as bridging technology.^18^

To test the stability of the doped oh-PtNi catalysts under appropriate conditions,^19^ we used the US DOE recommended accelerated stress test (AST) protocol,^20^ which includes 30,000 potential cycles between 0.6 and 0.95 V vs RHE with a hold time of 3 s at each potential limit, which is an AST protocol that corresponds to a predicted system lifetime of 5000 h.^21^

Here, we demonstrate high catalytic ORR reactivity and stability of doped octahedral oh-PtNi catalysts in a GDE half-cell system. We performed a square-wave AST protocol to evaluate and compare the stability of Mo- and MoRh-doped oh-PtNi catalysts with that of Pt/C, the benchmark catalyst. In addition, we analyzed the GDE catalytic layer before and after the stability tests to investigate Ni dissolution and particle agglomeration. Our study identifies new promising Pt alloy ORR catalyst candidates and their ink and layer preparation protocols for improved PEMFC cathodes.

## Experimental Section

### Materials

Platinum(II) acetylacetonate [Pt(acac)_2_] (98%) was purchased from Agros Organics. Nickel(II) acetylacetonate [Ni(acac)_2_] (≥98%) was purchased from Merck KGaA. Molybdenum hexacarbonyl (≥99.9% trace metals basis) was purchased from Sigma-Aldrich. Rhodium(III) acetylacetonate [Rh(acac)_3_] (Rh 25.2% min), benzoic acid (99%), and polyvinylpyrrolidone (PVP, M.W. 10,000) were purchased from Alfa Aesar. Benzyl alcohol (BA, ≥99%) was purchased from Carl Roth. Carbon Vulcan (XC72-R) was purchased from Cabot. For the washing, ethanol (VWR, absolute, ≥99.8%), acetone (VWR, absolute, ≥99.5%), and ultrapure water (Milli-Q, 18.2 MΩ) were used. All of the chemicals were used as received without any purification.

### Synthesis of PtNi(Mo)/C and PtNi(MoRh)/C Octahedral Nanoparticles

In a typical one-pot synthesis of Ni-rich octahedral-shaped nanoparticles (NPs), 64 mg of Pt(acac)_2_, 200 mg of Ni(acac)_2_, 20 mg of Mo(CO)_6_, in the case of Rh doping 20 mg of Rh(acac)_3_, 640 mg of PVP (M.W. 10,000), 400 mg of benzoic acid, and BA (60 mL) were added into a 100 mL pressure vessel with a stirring bar. The reaction mixture was heated from room temperature to 60 °C and kept for 1 h under vigorous stirring. At the same time in a glass vial with a magnetic stir bar, a suspension of 75 mg of Vulcan carbon XC72-R in 10 mL of BA was prepared and stirred overnight. The reaction solution was heated to 150 °C and kept for 12 h while stirring the mixture during the whole solvothermal treatment. The solution was allowed to cool to room temperature and the carbon suspension was added. The mixture was stirred overnight, followed by the washing procedure as described in the “Washing Procedure” section below.

### Washing Procedure

After the mixture was stirred overnight, the pressure vessel was opened, and the suspension was centrifuged in a 250 mL container. The particles were dispersed in 120 mL of a 2:1 solution of acetone and ethanol, and the centrifuge container was ultrasonicated (5–10 min). After centrifugation (10 min, 7500 rpm) of the suspension, solvents were discarded and the NPs were air-dried overnight.

### Characterization

X-ray powder diffraction (XRD) patterns were collected using a D8 ADVANCE diffractometer (Bruker) equipped with a LYNXEYE detector and a KFL Cu 2K X-ray tube. The measurement was carried out at a step size of 0.04°, in a 2θ range between 20 and 85°. The atomic composition of the different NPs was determined by inductively coupled plasma optical emission spectrometry (ICP–OES) using a 715-ES-ICP analysis system (Varian). Transmission electron microscopy (TEM) images were recorded on a FEI Tecnai G2 20 S-TWIN with a LaB_6_ cathode operating with 200 kV acceleration voltage and a resolution limit of 0.24 nm. Scanning electron microscopy (SEM) images were obtained using a Hitachi SU8030 instrument with a cold field emission gun operating with a 10 kV acceleration voltage. Corresponding energy-dispersive X-ray (EDX) spectroscopy analysis was established by an Ametek Inc., EDAX APEX2.5.

### Electrochemical Measurements

For the determination of electrochemical surface areas (ECSAs), activities, and stabilities of the catalysts, a RDE three-electrode cell setup in a liquid electrolyte with a glassy carbon (GC) working electrode (WE) was used. The electrochemical cell consisted of a Pt mesh as the counter electrode (CE), an RHE or MMS reference electrode (RE), and a GC as the WE. The WE was controlled by a rotator from Pine Research Instrumentation. Electrochemical measurements were performed with a BioLogics Science Instruments potentiostat model SP-150 and VSP. Potentials obtained with the MMS RE were converted to the RHE, and the potential difference between the MMS and the RHE was measured in a H_2_-saturated 0.1 M HClO_4_ solution. The catalyst ink for the RDE was prepared by ultrasonication of 1.8–3 mg of catalyst in a solution consisting of 79.6 vol % deionized water, 20 vol % isopropanol, and 0.4 vol % Nafion ionomer (5 wt %, Sigma-Aldrich). Then, 10 μL of the homogeneous ink was deposited on a polished GC-RDE, followed by drying at 60 °C. The Pt loading of the catalysts on the electrodes was 10 μg_Pt_ cm^–2^. The catalyst thin film was first activated by cyclic voltammetry (CV) from 0.05 to 0.925 V_RHE_ (50 cycles) at a scan rate of 100 mV s^–1^ in N_2_-saturated 0.1 M HClO_4_. The ECSA of the catalysts was determined by H_upd_- and CO-stripping experiments. The catalytic ORR activity was tested by using linear sweep voltammetry (LSV) from 0.05 to 1.0 V vs RHE at a scan rate of 20 mV s^–1^ in O_2_-saturated 0.1 M HClO_4_ with a rotation speed of 1600 rpm. All obtained potentials were corrected by using IR correction. The resistance (*R*) was determined by potentiostatic electrochemical impedance spectroscopy (PEIS). The AST was conducted by performing square-wave potential cycling from 0.6 to 0.95 V vs RHE for 30,000 cycles with a dwelling time of 3 s for each potential in N_2_-saturated 0.1 M HClO_4_. Further details of the RDE measurements are summarized in the Supporting Information.

### GDE Preparation and Testing

GDE characterization was performed using a commercial half-cell (FlexCell PTFE, Gaskatel GmbH) operated at room temperature in 2 M HClO_4_. The used cell consists of a gas chamber and an electrolyte chamber, which includes a separate reservoir for the RE (HydroFlex, Gaskatel GmbH), which is connected to the WE via a Luggin capillary. The utilized half-cell setup is a three-electrode cell that uses a RHE as the RE, a titanium expanded metal coated with Ir/Ta mixed oxide as the CE, and the GDE as the WE. Electrochemical measurements were carried out on an Ivium multichannel potentiostat (OctoStat 5000) which is controlled by IviumSoft software. The catalyst ink for the GDE was prepared by mixing 3 mg of catalyst with a 4:1 solution of deionized water and isopropanol and further mixed with Nafion ionomer (5 wt %, Sigma-Aldrich) to obtain an ionomer to carbon ratio (I/C) of 0.5 g/g. The total volume was 3 mL to get an ink with a concentration of 1 mg_catalyst_/mL_ink_. The ink was dispersed 3 times, 1 min each, by an ultrasonic processor (Hielscher, UP200 St). For GDE preparation, the catalysts were deposited on a gas diffusion layer (GDL, Sigracet 25 BC) by using a drop-casting method, resulting in catalyst loadings of 92, 42, and 26 μg_Pt_ cm^–2^ for HiSPEC 3000, PtNi(Mo)/C, and PtNi(MoRh)/C, respectively. Detailed information on the GDE preparation is given in Supporting Information. The GDE was pretreated by CV from 0.05 to 1.2 V vs RHE (200 cycles) at a scan rate of 500 mV s^–1^ in a N_2_ atmosphere to activate the catalyst. The ECSA of the catalysts was determined by H_upd_- and CO-stripping experiments. The ORR activity was measured by conducting a protocol consisting of galvanostatic steps. For the construction of the polarization curves, the average of the last five points (2.5 s) of each step is used. The uncompensated resistance (IR drop) in the used GDE setup was determined by using EIS and was used for correction of all measured potentials. For each experiment, at least two samples were tested. An overview on the applied measurement protocol is given in Table S2. A square-wave cycling between 0.6 and 0.95 V vs RHE with 3 s holding time was carried out for a total of 30,000 cycles. Before the test and after a total number of 5000, 15,000, and 30,000 cycles, a complete electrochemical characterization was performed according to Table S2. The AST was performed under a N_2_ atmosphere and 2 M HClO_4_.

## Results and Discussion

### Physicochemical Characterization

**Figure 1:**
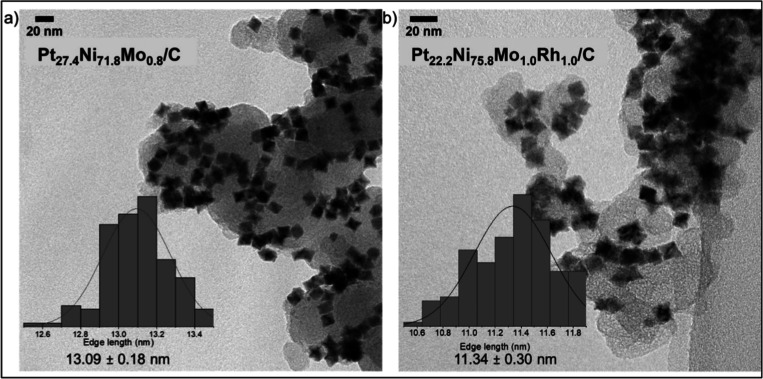
Bright-field TEM images of (a) carbon-supported Ni-rich Mo-doped oh-PtNi NPs (Pt_27.4_Ni_71.8_Mo_0.8_) and (b) carbon-supported Ni-rich MoRh-doped oh-PtNi NPs (Pt_22.2_Ni_75.8_Mo_1.0_Rh_1.0_); insets: edge length histograms and distribution function and mean edge length (see Table S3 for additional compositional information).

A number of different Mo- and RhMo-doped octahedrally shaped oh-PtNi catalysts were synthesized using a solvothermal protocol published elsewhere.^22,23^ The syntheses resulted in Ni-rich Mo- or MoRh-doped oh-NPs with mean edge-length particle sizes of 13.1 and 11.3 nm, respectively. Particle sizes were manually measured using acquired TEM (Figure 1). Nominal nickel and platinum ratios as well as the actual resulting Pt/Ni atomic ratios of the NPs are provided in Table S3. The X-ray diffraction patterns of the Ni-rich Mo- or MoRh-doped oh-PtNi NPs are shown in Figure S2, where the reflections shifted to lower 2θ angles with increasing platinum content.

### Electrochemical Characterization Using RDE Half-Cells

**Figure 2:**
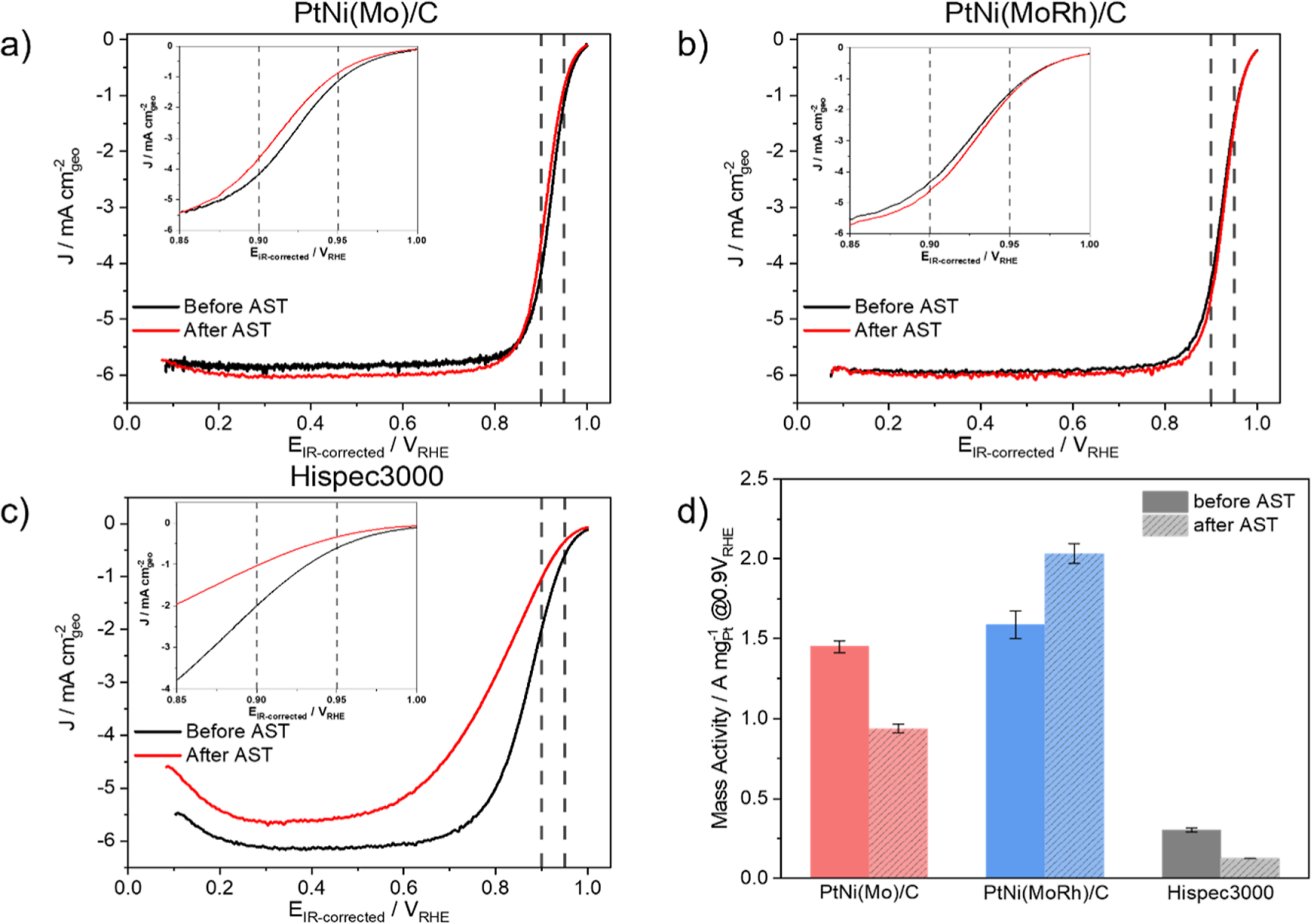
Electrochemical polarization curves and MAs of activated electrocatalysts obtained by the RDE half-cell. ORR LSVs of (a) Mo-doped oh-PtNi, (b) MoRh-doped oh-PtNi, (c) Pt/C HiSPEC 3000, and the (d) corresponding MAs at 0.9 V vs RHE evaluated before and after the AST (30,000 cycles between 0.6 and 0.95 V vs RHE), calculated according to the Koutecký–Levich equation.

We used CV in the RDE setup to obtain electrochemical H_upd_- and CO-based stripping charges and the corresponding ECSAs of the oh-Pt alloy catalysts in N_2_- or CO-saturated electrolytes, respectively (Figure S3). We also determined the catalytic reactivity for the ORR using LSV in the O_2_-saturated electrolyte (Figure 2a–c). The H_upd_-based ECSAs were, as expected and well-documented, lower than the values obtained from CO-stripping measurements. Also, the H_upd_- and CO-based ECSA values of the HiSPEC 3000 Pt/C benchmark catalyst were, as expected, larger than those of the oh-Pt alloy catalysts, with the variance between the two getting smaller after the ASTs (Table 1) suggesting gradual non-noble transition metal leaching. Indeed, the catalyst morphology after the AST showed a hexapod structure, which is the result of Ni leaching from the octahedron facets (Figure S4).^24^

**Table 1:** Overview of the H_upd_- and CO-Based ECSA (RDE) before and after the AST (30k)

sample	ECSA_H_upd_-based_ [m^2^/g_Pt_]		ECSA_CO-based_ [m^2^/g_Pt_]	
	before AST	after AST	before AST	after AST
PtNi(Mo)/C	33.33	27.02	42.16	33.74
PtNi(MoRh)/C	39.57	38.04	52.43	44.54
HiSPEC 3000	68.31	47.49	69.46	52.32

After a Koutecký–Levich mass transport correction (eq 4, Supporting Information), we normalized the LSV currents obtained at 0.9 V vs RHE with the Pt mass deposited on the WE to obtain the mass activities (MAs) for the different catalysts before and after the AST (Figure 2d). Both the Mo- and MoRh-doped oh-PtNi catalysts exhibited 4.8- and 5.3-fold higher initial MAs compared with the commercial reference Pt/C (0.3 A mg_Pt_^–1^), respectively. After the AST, the Mo-doped oh-PtNi catalyst retained 73% of its initial MA (1.45 A mg_Pt_^–1^), while the MoRh-doped catalysts exhibited even increased MA reaching 2.03 A mg_Pt_^–1^, a unique property for Rh-doped oh-PtNi catalysts.^23,25^

### Electrochemical Characterization Using a GDE Half-Cell Setup

Gas diffusion electrons were prepared by spraying catalyst inks onto porous GDLs with microporous layers (Figure S18). To control the active spray-coated electrode surface area to precisely 0.5 cm^2^ on the GDL, we utilized custom-made PTFE masking schemes. To determine the precise catalyst loading on the GDE with a 0.5 cm^2^ coated area after removing the PTFE mask, we used the ratio of CO-based ECSAs with and without masking (Table S4).^26^ The H_upd_- and CO-based ECSAs obtained from the GDE were comparable to values acquired by the RDE and showed the same trends of decreasing ECSA during and after the ASTs (Figures S3, S5, and S6 and Table S5). Consistently, the ECSA values based on CO stripping were higher compared to the values based on H_upd_ for all catalysts tested.^15,27^

**Figure 3:**
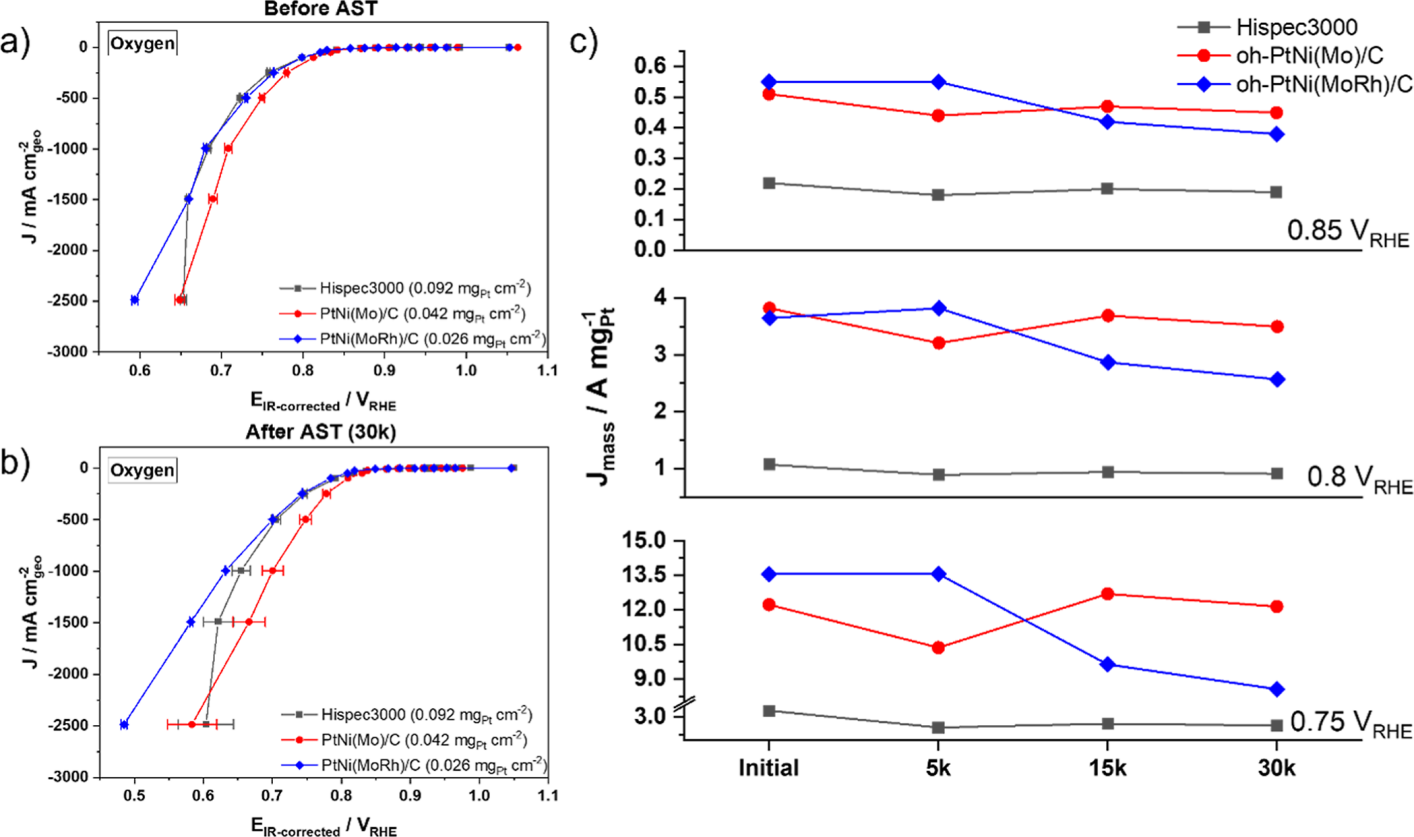
GDE half-cell measurements: electrochemical polarization curves and extracted Pt MAs of oh-Pt alloy electrocatalysts obtained in the GDE half-cell setup. ORR polarization curves of Mo-doped oh-PtNi, MoRh-doped oh-PtNi, and Pt/C HiSPEC 3000 (a) before the AST, (b) after the AST, and the (c) corresponding calculated after 5k, 15k, and 30k cycles at 0.75, 0.8, and 0.85 V vs RHE.

To obtain the polarization curves for the different catalysts with the GDE flow cell setup, we used a galvanostatic method, as this is a widely used electrochemical method for fuel cell testing. Also, this method has been shown to lead to better comparability between different catalyst loadings and thus different catalysts compared to ORR test protocols with potentiostatic control.^26,28,29^ Typical geometric Pt mass loadings on the GDE ranged around 0.026–0.092 mg_Pt_ cm^–2^, compared to 0.01 mg_Pt_ cm^–2^ in the RDE. Catalytic ORR activity became apparent for all catalysts cathodic of 0.9 V vs RHE (Figure 3). This onset potential was slightly more cathodic compared to those obtained in RDE setups, likely due to inhibition by the much higher concentrated perchloric acid.^30^ Accordingly, the initial MAs of the oh-Pt alloy catalysts obtained from RDE tests at a cathode potential of 0.9 V vs RHE ranged higher than those obtained in the GDE setup @0.85 V vs RHE (0.1 and 2 M perchloric solution, respectively, Figures 2d and 3c). Clearly, the GDE setup was able to achieve ORR current densities in the A cm^–2^ range and therefore represented a much more relevant and predictive electrode performance for PEM fuel cell membrane electrolyte assemblies. Throughout all electrode potentials, the shaped Pt alloy catalysts exhibited much higher MAs than the Pt/C benchmark catalysts (Figures 2 and 3). In addition, the shaped catalysts outperformed the Pt/C catalysts in terms of stability during and after the ASTs and exceeded the catalytic activity of the Pt/C catalysts at every interval of 5000 stability cycles. For example, the Pt/C MA @0.75 V vs RHE lacked behind that of the shaped catalysts @0.8 V vs RHE. After the AST, the Mo-doped oh-catalyst retained most of its initial MA, while MoRh-doped catalysts lost about 30% of it @0.8 V vs RHE. After the galvanostatic steps in oxygen, we also tested the oh-Pt alloy catalysts with synthetic air feed (see Table S2 for the testing protocol). The catalysts showed the same activity and stability trends but at lower absolute values, as expected for a lower partial pressure oxygen feed (Figure S9). At potentials less than 0.65 V vs RHE, the shaped oh-Pt alloy catalysts began to lose their advantages over the Pt/C catalyst, probably due to mass transport limitations and low Pt loadings.^28^

Important stability differences between RDE and GDE testing became apparent as well. The RDE results predicted that the MoRh-doped oh-Pt alloy catalyst showed a higher MA at 0.9 V vs RHE both before and after the AST compared to the Mo-doped catalyst (Figure 2d). By contrast, the GDE results at 0.85, 0.8, and 0.75 V vs RHE revealed an opposite trend after a 15,000-cycle AST (Figure 3c). This could indicate that the MoRh-doped catalyst degraded faster than the Mo-doped catalyst under GDE conditions. One reason for that could be distinct Ni-leaching rates due to the higher (2 M) acid concentration in the GDE.

**Figure 4:**
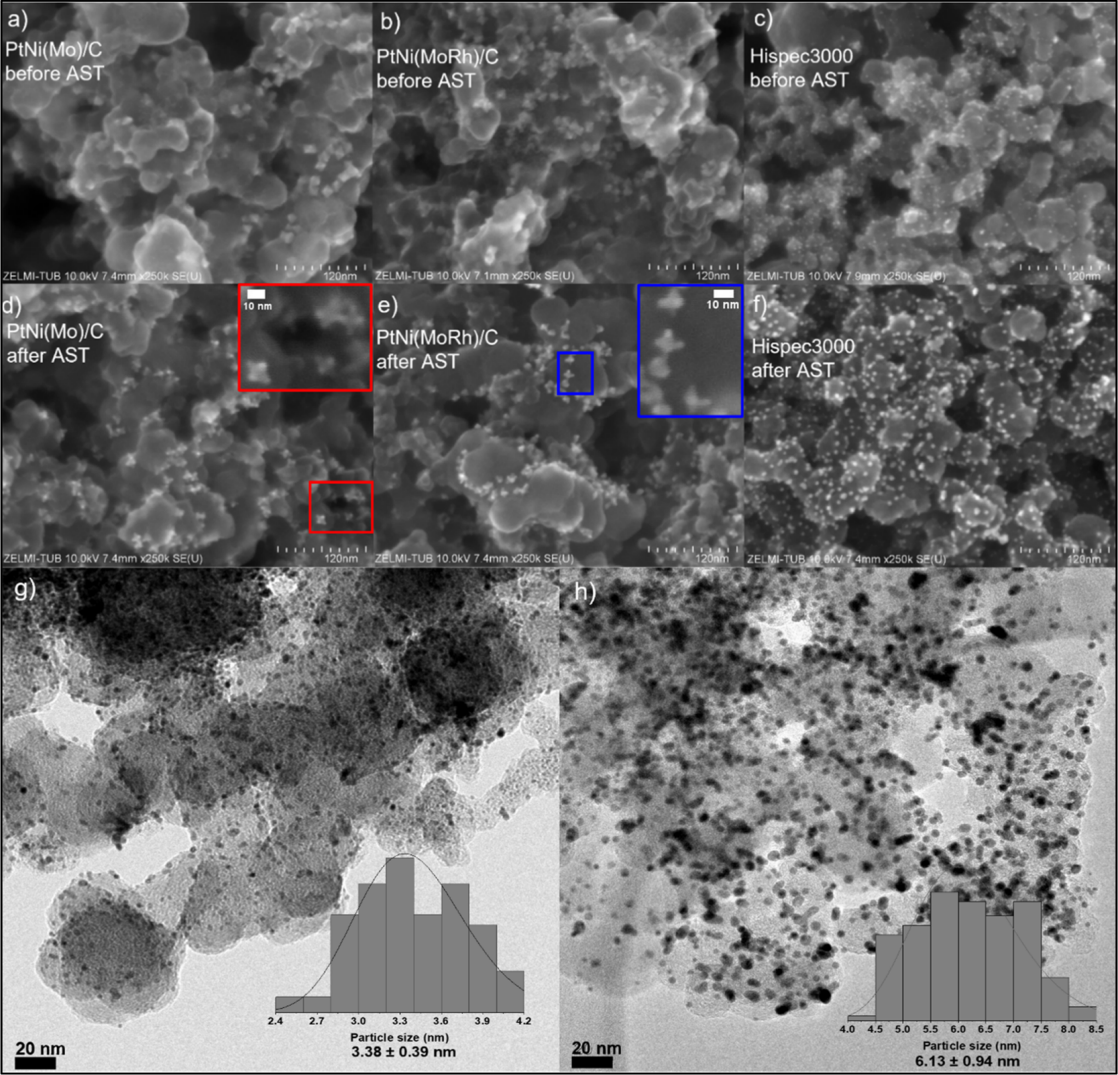
SEM images with a magnification of 250k of the as-prepared (a) Ni-rich Mo-doped oh-PtNi NPs, (b) Ni-rich MoRh-doped oh-PtNi, and (c) Pt/C HiSPEC 3000 catalysts collected with the secondary electron detector and of the corresponding tested catalysts (after the AST) in (d–f), respectively. The colored insets in panels (d,e) highlight the zoomed-in views of the aged Mo- (red) and MoRh-doped (blue) oh-PtNi NPs. Bright-field TEM images of Pt/C HiSPEC 3000 NPs (g) before and (h) after the AST and the particle size distribution in the insets.

As done for RDE-tested electrodes, we used SEM to investigate the morphology of the catalyzed GDE before and after ASTs. We found that after the AST, the morphology of the initially oh-shaped doped PtNi/C catalysts turned into a hexapod structure, obviously a result of significant Ni leaching from the octahedral facets (Figure 4a,b,d,e). The Ni leaching was also confirmed by the EDX spectroscopy measurements on the catalysts, which showed that the Pt/Ni ratio increased after ASTs (Figures S10–S17). The back-scattered image evidences the characteristic Ni-leached multipod morphology (Figure S19).

Noteworthily, the oh-shaped doped PtNi/c catalysts did not exhibit apparent particle agglomeration (Figures S19 and S20), while the Pt/C catalyst increased its mean particle size from 3.4 to 6.1 nm due to particle agglomeration or Ostwald ripening, as shown in Figure 4c,f–h. These observations are in line with similar reports^31^ based on identical location-TEM techniques. The increase in Pt/C particle size is supported by the relatively steep decrease in its ECSA (Figures S5 and S6).

Since testing catalysts for ORR using a GDE half-cell setup are still poorly used or reported, there are only a few comparable reports in the literature, especially for bimetallic catalysts.^11,15,16,26,32^ While the Pt/C catalyst had comparable activity values to previously published GDE results, GDE-based stability data of oh-shaped Pt-based alloy catalysts have never been reported before. However, the MEA-based activity of doped octahedral PtNi catalysts was published earlier. Dionigi et al.^33^ reported RDE activity more than 7 times higher than MEA activity (3.43 and 0.45 A mg_Pt_^–1^ @0.9 V vs RHE, respectively). Pan et al.^34^ reported a MEA-based MA of 0.35 A mg_Pt_^–1^ at 0.9 V vs RHE but a significantly high performance at high current density for octahedral PtNi NPs, 1500 mA cm^–2^ at 0.6 V vs RHE, to our knowledge the highest to date for this class of catalysts.

Given the more realistic testing environments, the larger current densities, and the prevention of oxygen mass transport limitations in the GDE setup, we consider our obtained catalytic performance and stability results as much more reliable and predictive for single PEM fuel cell testing compared to conventional RDE test data.

## Conclusions

We report and discuss a comparative investigation of the catalytic ORR performance and stability of carbon-supported shape-controlled Mo- and MoRh-doped oh-PtNi/C catalysts in conventional RDE half-cells and GDE half-cells. The ORR activity and stability were tested in RDE half-cell and GDE flow half-cell setups in (RDE typical) lower and (GDE typical) higher overpotential (and hence current density) regions, respectively. The shaped doped Pt alloy catalysts showed superior ORR activity compared to the Pt/C benchmark catalyst over a wide range of working potentials from 0.65 to 0.85 V vs RHE. We tested the catalyst stability using a recent DOE-issued square-wave stability test. In doing so, we found that the octahedral catalysts maintained their excellent activity throughout the stability test and after 30,000 stability cycles. While the Pt/C catalyst showed agglomeration accompanied by a sharp decrease in its ECSA, the octahedral catalysts did not show significant particle agglomeration, and only a slight decrease in their ECSAs was observed. Given the larger current densities, the GDE test results feature a more reliable predictive character for their electrode performance in full PEM fuel cells compared to RDE tests, in particular, for catalyst stability under fully humidified conditions, where incorrect RDE-based stability predictions of PEMFC results are common. Our electrochemical performance data of Pt-based oh-Pt alloy catalysts at low Pt loadings are a testament to exceptionally high Pt MAs over a wide range of electrode potentials and current densities (several A cm^–2^ range) combined with excellent stability. While these results provide new specific catalyst candidates for deployment at cathodes in PEMFCs, our comparative approach highlights the practical value of advanced shaped catalysts in both the low- and high-current-density regimes, the latter of which has been underutilized to date.

## Supporting Information Available

Detailed information on the synthesis, chemical, and structural characterization of the catalyst by ICP–OES, TEM, and XRD; electrochemical performance and stability protocols, as well as the data obtained by RDE and GDE half-cell experiments; and SEM images and EDX data of the GDEs before and after ASTs (PDF)
