# Supplementary material for: Oxygen Reduction Reaction Activity and Stability of Shaped Metal-Doped PtNi Electrocatalysts Evaluated in Gas Diffusion Electrode Half-Cells
Source: ACS Appl Mater Interfaces. 2024 Sep 19;16(39):52406–13. doi: 10.1021/acsami.4c11068 (PMC11450720; doi:10.1021/acsami.4c11068)
Supplement: Supplementary file 1 — am4c11068_si_001.pdf [file am4c11068_si_001.pdf]

Supporting Information

Oxygen Reduction Reaction Activity and Stability of Shaped Metal-Doped PtNi Electrocatalysts evaluated in Gas Diffusion Electrode Half-Cells

Shlomi Polani[a†,*], Raffaele Amitrano[a†], Adrian Felix Baumunk[b], Lujin Pan[a], Jiasheng Lu[a], Nicolai Schmitt[d], Ulrich Gernert[c], Malte Klingenhof[a], Sören Selve[c], Christian M. Günther[c], Bastian J. M. Etzold[b], and Peter Strasser[a,*]

[a]*Electrochemical Energy, Catalysis and Material Science Laboratory, Department of Chemistry, Technical University Berlin, 10623 Berlin, Germany.*

[b] *Friedrich-Alexander-Universität Erlangen Nürnberg, Power-to-X Technologies, Dr.-Mack-Straße 81, 90762 Fürth, Germany.*

[c]*Center for Electron Microscopy (ZELMI), Technical University of Berlin, Berlin, Germany.*

[d]*Ernst-Berl-Institute for Technical Chemistry and Macromolecular Science, Technical University of Darmstadt, Peter-Grünberg-Strasse 8, 64287, Darmstadt, Germany.*

†These authors contributed equally.

***Corresponding Authors**

Peter Strasser: pstrasser@tu-berlin.de

Shlomi Polani: shlomip@ariel.ac.il

Physico-chemical Characterization

ICP-OES preparation and evaluation. Each sample was prepared by dissolving the nanoparticles powders in a mixture of H_2SO_4, HNO_3 and HCl (1:1:3). The solutions were heated from room temperature to 180°C in 10 min using a Microwave Discover SP-D (CEM corporation), keeping at this temperature for another 20 min. The cooled solutions were diluted with MilliQ water, filtered, and taken to a known volume.

To estimate the concentration of each solution, 4 standards of Pt, Ni, Mo and Rh with a concentration of 1, 7, 12 and 20 mg/L of each element were prepared. The concentration of the calibration solutions was 1000 mg/L for Pt (Certipur®) and Ni (Carl Roth), 1001 mg/L for Rh, as well as 999 mg/L for Mo (both Fluka Analytical).

For the calculation of the composition of the samples the concentration at different wavelengths was used, which can be seen in Table S1.

Table S1: Wavelengths used to determine the concentrations for Pt, Ni, Mo and Rh via ICP-OES.

Element	Wavelength λ [nm]			
Pt	306.471			
Ni	216.555	230.299	231.604	232.138
Mo	201.512	202.032		
Rh	249.078	343.488		

TEM preparation and evaluation. For sample preparation, the particles were dispersed in ethanol with ultrasonication (5 min), deposited on a Cu grid (300 mesh), and air-dried. The edge length of the particles was determined from the TEM images by counting of at least 150 particles.

SEM evaluation. The particle size of the reference catalyst Hispec3000 was determined from the SEM images before and after AST by counting of at least 150 particles.

Electrochemical measurements

Rotating disk electrode preparation. The GC electrodes were polished with an alumina polish of 1.0 µm particle size (Buehler Alpha) on a Nylon sheet for 2 min and subsequently, with an alumina polish of 0.05 µm particle size (Buehler Alpha) on a MicroCloth sheet for 2 min. The electrodes were then sonicated in ultrapure water, absolute acetone, and absolute ethanol for 5 min each to obtain a clean surface.

Electrolyte preparation. A 0.1 M $HClO_4$ electrolyte solution was prepared by diluting 4.32 mL of 70 % redistilled perchloric acid in ultrapure water to reach a volume of 500 mL. The electrolyte was stored in an opaque bottle.

Ink preparation. To prepare the ink for the RDE measurements, a certain mass of catalyst (approx. 1.8−3 mg) was added to a solution consisting of 79.6 Vol-% deionized water, 20 Vol-% isopropanol, and 0.4 Vol-% Nafion™ ionomer (5 wt%, Sigma-Aldrich). The exact volume depended on the amount of catalyst and the platinum loading on the carbon support. The area of the GC RDE was 0.196 cm^2. To achieve a Pt loading of 0.010 mg_{Pt} cm^{-2} the volume was determined by the following equation (1).

$$V_{total} = \frac{m \cdot 10\ \mu L \cdot wt\%(Pt)}{100\ g \cdot 1.96\ \mu g} \quad (1)$$

The suspension was then sonicated with an ultrasonic horn sonicator (Branson Sonifier 150) for 30 min while immersed in an ice bath. The ink was used on the same day. 10 µl of the homogeneous ink were pipetted on a GC-RDE (diameter Ø = 5 mm). The thin film was dried at 60°C for electrode preparation.

Cell cleaning and preparation. To clean the electrochemical cell, it was rinsed with ultrapure water and then dried in an oven at 60°C. Before every use, the cell was flushed and then filled with the electrolyte. The platinum counter electrode was also flushed with electrolyte and inserted into the cell. The reference electrode was cleaned with ultrapure water and then flushed with electrolyte before insertion into the cell. The degree of purity for the used gases was 99.998 % for oxygen and 99.999 % for nitrogen.

Electrochemical Characterization, CO stripping, activity, and stability test using RDE. To evaluate the electrochemical surface area (ECSA) and the activity we perform the measurements in the order suggested by Rudi et al.[1] To avoid the effect of CO-stripping on the catalyst, two working electrodes were used to obtain CO-stripping based on ECSA before and after the accelerated stress test (AST). Figure S1 presents the flow chart of the different steps applied to two identical working electrodes. The initial pathway was conducted three times on each catalyst, whereas the pathway including the stability test was repeated once on each catalyst.

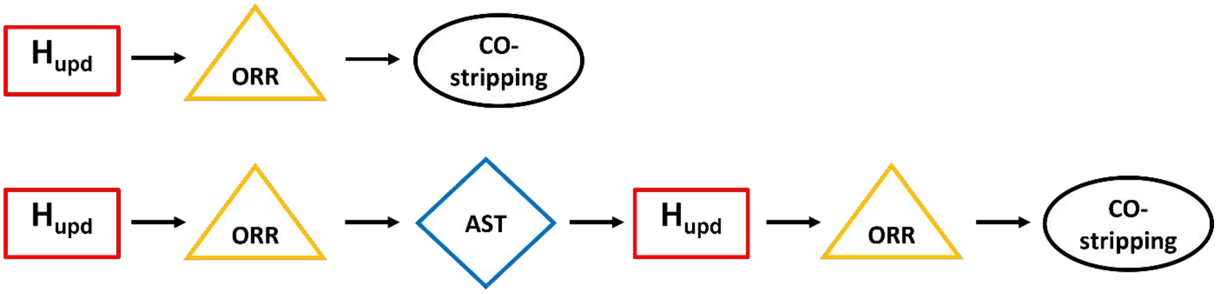

Figure S1: Detailed RDE testing protocols of the electrochemical characterization.

Activation step and H_{upd}-based ECSA. Cyclic voltammetry (CV) in the potential range from 0.05−0.925 V_{RHE} (50 cycles) with a scan rate of 100 mV s^{-1}, in N_2-saturated 0.1 M $HClO_4$ was performed to obtain a stable voltammogram. After activation three CV cycles in the same potential range were applied with a scan rate of 20 mV s^{-1}. The third cycle was used to determine the H_{upd}-based ECSA by integrating the hydrogen adsorption charge from approx. 0.05−0.4 V_{RHE}. The resistance (R) was determined by potentiostatic electrochemical impedance spectroscopy (PEIS) after applying a potential of 0.5 V_{RHE} for 5 minutes.

ORR Testing. Before the ORR measurements were performed in O_2-saturated 0.1 M $HClO_4$, the background current was determined in N_2-saturated 0.1 M $HClO_4$. The activity of the catalyst was extracted using linear sweep voltammetry (LSV) in the potential range from 0.05–1.0 V_{RHE} (anodic scan) with a rotation speed of 1600 rpm. The scan rate was 20 mV s^{-1}. The scan was repeated two times.

CO-stripping-based ECSA. To obtain the ECSA based on CO-stripping, the WE was inserted in the N_2-saturated electrolyte with a rotation speed of 400 rpm at 0.05 V_{RHE}. After CO gas was bubbled for 30 seconds, N_2 was bubbled into the solution for at least 10 minutes to remove the dissolved CO. Subsequently, in a stationary mode, the CV was recorded between 0.05–1.1 V_{RHE} with 50 mV s^{-1} for 3 cycles. The CV of the reference catalyst Hispec3000 was recorded from 0.05–1.2 V_{RHE} with 50 mV s^{-1} for 3 cycles. The ECSA was determined based on Q_{CO}, which was obtained by integrating the area of the CO-stripping peak.

Accelerated Stress Test. The AST was performed in an N_2-saturated 0.1 M $HClO_4$. Square wave potential cycling was conducted from 0.6 to 0.95 V_{RHE} for 30,000 cycles with a dwelling time of 3 s for each potential.

Data Correction. For LSV and CV measurements the potential was corrected to the value on the RHE scale (equation (2)).

$$\mathrm{E_{RHE} = E_{measured} + E_{calibration}} \tag{2}$$

The resulting potential E_{RHE} was IR corrected with the resistance R obtained from PEIS (equation (3)).

$$\mathrm{E = E_{RHE} - I \cdot R} \tag{3}$$

For the background correction of the current I_{ORR}, the current of the N_2-saturated LSV was subtracted from the current of the oxygen saturated LSV (equation (4)).

$$\mathrm{I_{ORR} = I_{O_2} - I_{N_2}} \tag{4}$$

Gas Diffusion Electrode. For the electrochemical characterization by a gas diffusion electrode (GDE) a commercial half-cell (FlexCell® PTFE, Gaskatel GmbH) was used at room temperature. The electrolyte used was perchloric acid (ROTIPURAN®Ultra 70%, Carl-Roth), that was diluted with deionized water (< 1.1 µS cm^{-1}, VWR chemicals) to a solution with a concentration of 2 M. While the electrolyte can only be flushed with N_2 (99.99%, Air Liquide), the gas compartment can separately be purged with either N_2, O_2 (99.99%, Air Liquide), CO (99.99%, Air Liquide), H_2 (99.99%, Air Liquide) or synthetic air (20.5% O_2 in N_2, Air Liquide).

GDE preparation. The used gas diffusion layer (GDL, Sigracet 25 BC) is sandwiched between an aluminum block, which is preheated to 125 °C by a heating plate and a PTFE mask. The PTFE mask has a hole with an area of 0.5 cm^2 where 255 µL of the catalyst ink is pipetted into and dried at 125 °C. During the drying some of the catalyst ink sticks to PTFE mask and is not applied onto the GDL. To account for those losses of catalyst, a second GDL is prepared the same way, but without the PTFE mask to ensure full deposition of the catalyst. The exact loading is determined from the ECSA from CO-stripping by using equation (5).

$$\mathrm{Catalyst\ loading_{real} = Catalyst\ loading_{nominal} \cdot \frac{ECSA_{CO,\ w\ ith\ mask}}{ECSA_{CO,\ without\ mask}}} \tag{5}$$

The nominal catalyst loading was 0.1 mg_{Pt} cm^{-2} for a Pt loading of 20 wt% and 0.09 mg_{Pt} cm^{-2} for a Pt loading of 18 wt% on carbon.

H_{upd}- and CO-stripping-based ECSA. The $ECSA_{Hupd}$ was determined by the integration of the hydrogen desorption area (H_{upd}) of the conducted CVs (3 cycles) in the same potential range at a scan rate of 100 mV/s.

For the ECSA based on CO-stripping CVs (3 cycles) were conducted in a potential range of 0.1−1.2 V_{RHE} at a scan rate of 20 mV/s. The $ECSA_{CO}$ was determined by the integration of the area of the CO-stripping peak.

ORR activity. The ORR protocol started with the reduction of the Pt surface by applying a potential of 0.1 V_{RHE} for 5 min. Then, the galvanostatic steps with different hold times were conducted with EIS.

Stability test. The stability test is based on the U.S. Department of Energy (DOE) protocol.[2]

Table S2: Protocol of the electrochemical characterization by GDE.

	**Step**	**Electrochemical Method**	**Parameters**	
**1**	RE potential	OCV	Gas purge and Time	H_2 (200 ml min^{-1}) for 200 s
**2**	Pretreatment	CV	Gas purge and Time	N_2 (300 ml min^{-1}) for 5 min
			Potential limits	0.05 – 1.2 V vs. RHE
			Scan rate	500 mV s^{-1}
			Number of cycles	200
**3**	ECSA (H_{upd})	CV	Gas purge and Time	N_2 (300 ml min^{-1}) for 5 min
			Potential limits	0.05 – 1.2 V vs. RHE
			Scan rate	100 mV s^{-1}
			Number of cycles	3
**4**	Oxide reduction	CA	Gas purge and Time	N_2 (300 ml min^{-1}) for 5 min
			Potential	0.1 V vs. RHE
**5**	Polarization curve (O_2)	OCV	Gas purge and Time	O_2 (200 ml min^{-1}) for 10 min
		Galvanostatic steps with EIS	Gas purge	O_2 (200 ml min^{-1})
			Current steps (Hold time)	-0.05/ -0.1 mA cm^{-2} (90 s)
				-0.25/ -0.5 mA cm^{-2} (60 s)
				-1/ -2.5/ -5 mA cm^{-2} (30 s)
				-25/ -50/ -100/ -250 mA cm^{-2} (5 s)
				-0.5/ -1/ -1.5/ -2.5 A cm^{-2} (5 s)
				Same steps back to -0.05 mA cm^{-2}
			EIS frequency range	F = 200 kHz – 10 Hz
			EIS amplitude	10 % of current (min. 5 mA cm^{-2})
**6**	Polarization curve (Syn. Air)	OCV	Gas purge	Syn. Air (200 ml min^{-1}) for 10 min
		Galvanostatic steps with EIS	Gas purge	Syn. Air (200 ml min^{-1})
			Current steps (Hold time)	-0.05/ -0.1 mA cm^{-2} (90 s)
				-0.25/ -0.5 mA cm^{-2} (60 s)
				-1/ -2.5/ -5 mA cm^{-2} (30 s)
				-25/ -50/ -100/ -250 mA cm^{-2} (5 s)
				-0.5/ -1/ -1.5/ -2.5 A cm^{-2} (5 s)
				Same steps back to -0.05 mA cm^{-2}
			EIS frequency range	F = 200 kHz – 10 Hz
			EIS amplitude	10 % of current (min. 5 mA cm^{-2})
**7**	ECSA (CO-Stripping)	CA	Gas purge and Time	CO (200 ml min^{-1}) for 2 min
			Potential	0.1 V vs. RHE
			Gas purge	N_2 (500 ml min^{-1}) for 45 min
			Potential	0.1 V vs. RHE
		CV	Gas purge and Time	N_2 (500 ml min^{-1}) for 5 min
			Potential limits	0.1 – 1.2 V vs. RHE
			Scan rate	20 mV s^{-1}
			Number of cycles	3

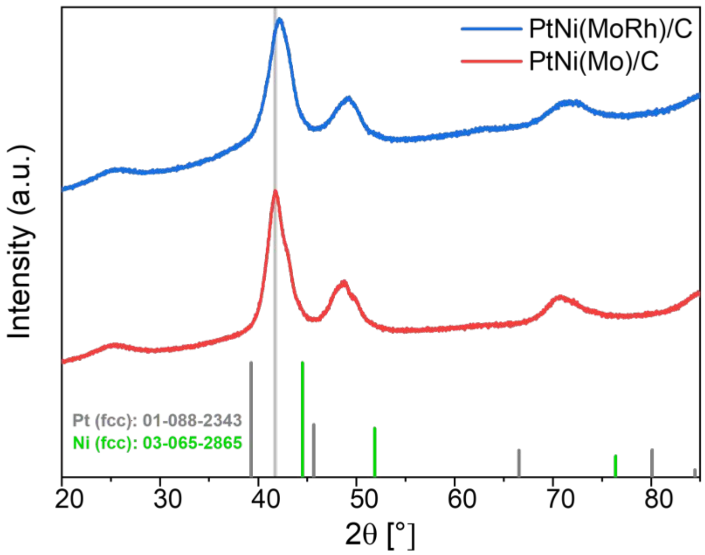

Figure S2: XRD patterns of PtNi(Mo)/C (red) and PtNi(MoRh)/C (blue). The grey lines correspond to pure Pt fcc (PDF No. 01-088-2343) and the green lines to pure Ni fcc (PDF No. 03-065-2865) patterns.

Table S3: ICP-OES results of the actual chemical molar compositions of the synthesized ternary and quaternary Pt-based octahedral nanoparticles.

Sample	Metallic at%	PtNi ratio	Pt wt%	Ni wt%	Mo wt%	Rh wt%
PtNi(Mo)/C	$Pt_{27.4}Ni_{71.8}Mo_{0.8}$	0.38	20.0	15.8	0.31	–
PtNi(MoRh)/C	$Pt_{22.2}Ni_{75.8}Mo_{1.0}Rh_{1.0}$	0.29	18.0	18.5	0.41	0.46

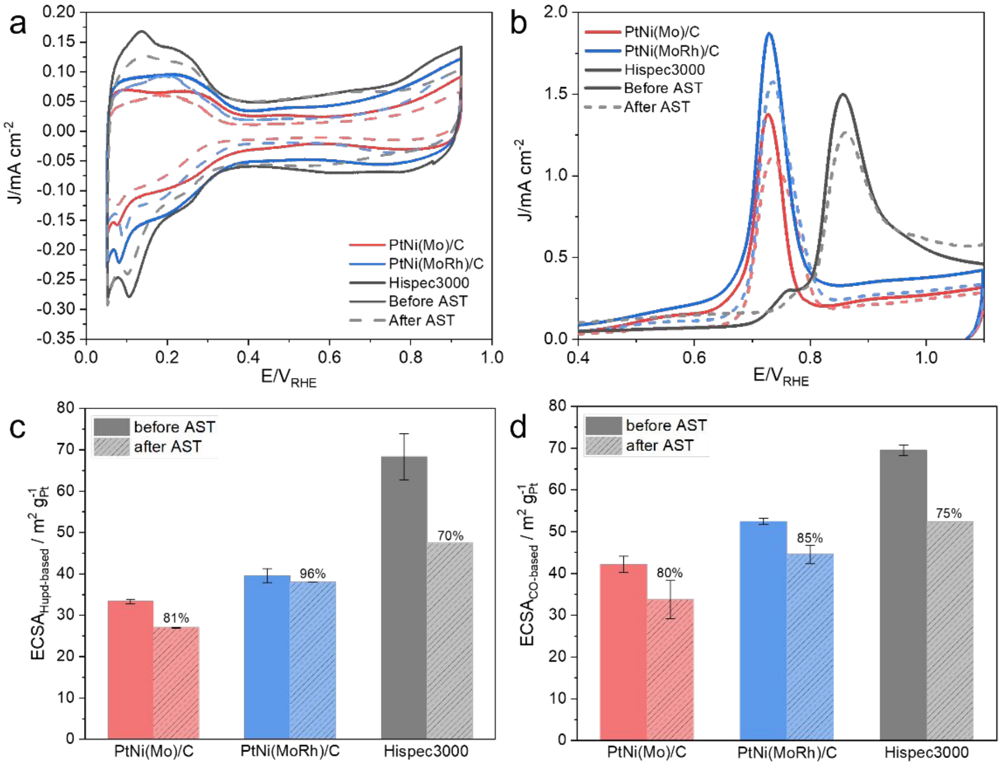

Figure S3: Electrochemical characterization in RDE (iR corrected) of PtNi(Mo)/C (red), PtNi(MoRh)/C (blue) and Hispec3000 (grey): a) cyclic voltammograms, b) CO-stripping voltammograms in N_2 saturated 0.1M $HClO_4$ electrolyte before (solid) and after AST (dashed), and the corresponding ECSAs c) based on H_{upd} and d) based on CO-stripping.

Table S4: Nominal and real catalyst loading on the GDL and the CO-based ECSA ratio with and without PTFE mask.

Sample	Pt wt%	Nominal catalyst loading on the GDL	$ECSA_{CO}$ ratio with and without PTFE mask	Real catalyst loading on the GDL
PtNi(Mo)/C	20.0	0.10 mg_{Pt} cm^{-2}	0.42	0.042 mg_{Pt} cm^{-2}
PtNi(MoRh)/C	18.0	0.09 mg_{Pt} cm^{-2}	0.29	0.026 mg_{Pt} cm^{-2}
Hispec3000	20.0	0.10 mg_{Pt} cm^{-2}	0.92	0.092 mg_{Pt} cm^{-2}

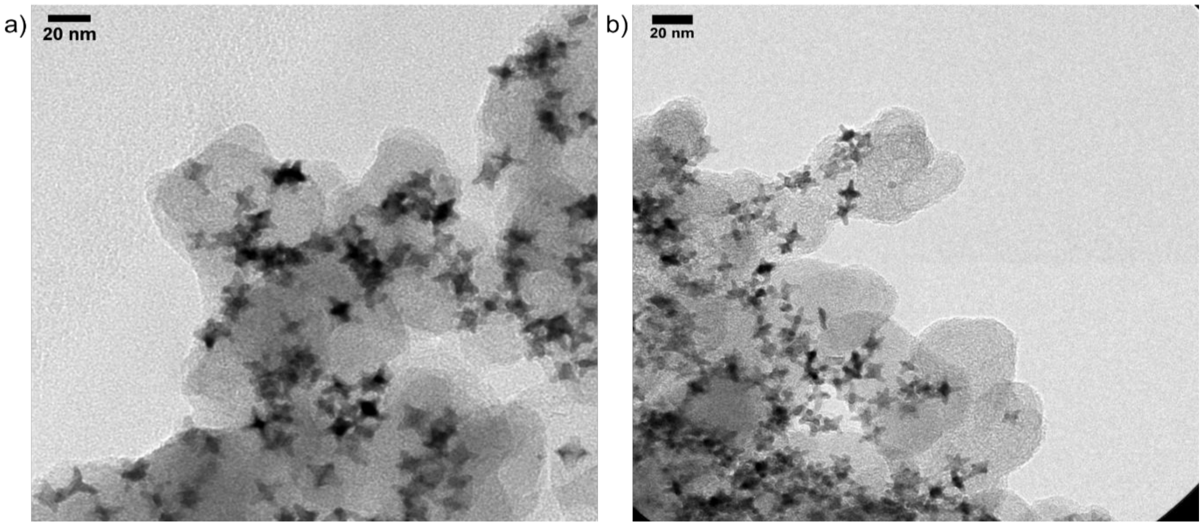

Figure S4: TEM images of a) PtNi(Mo)/C and b) PtNi(MoRh)/C nanoparticles after AST (30k).

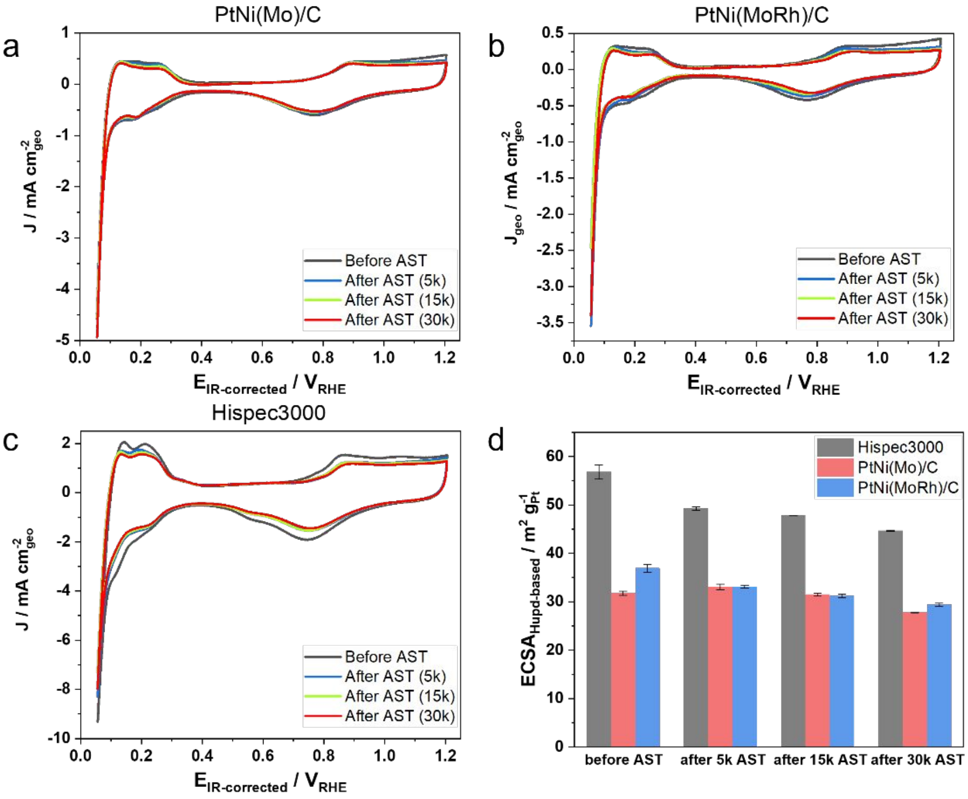

Figure S5: Cyclic voltammograms (iR corrected) of a) PtNi(Mo)/C, b) PtNi(MoRh)/C, and c) Hispec3000 in N_2 saturated 2M $HClO_4$ electrolyte before and after 5k, 15k, and 30k cycles AST and d) the ECSA based on H_{upd} in GDE.

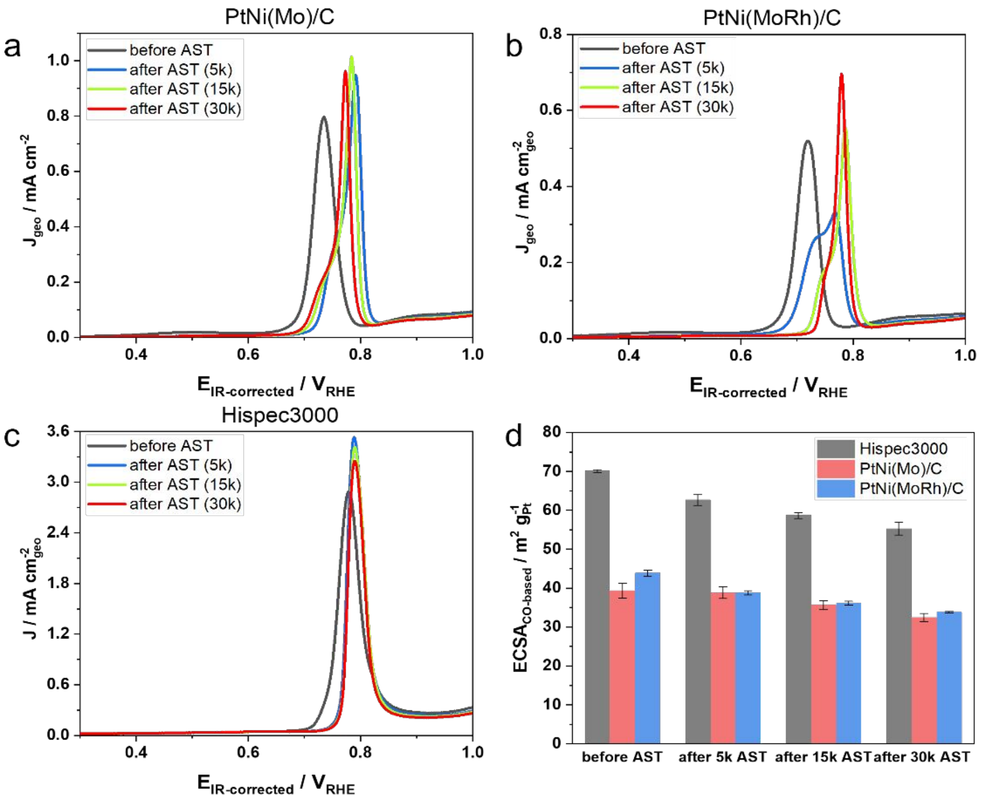

Figure S6: CO-stripping voltammograms (iR corrected) of a) PtNi(Mo)/C, b) PtNi(MoRh)/C, and c) Hispec3000 in N_2 saturated 2M $HClO_4$ electrolyte before and after 5k, 15k, and 30k cycles AST and d) the ECSA based on CO-stripping in GDE.

Table S5: Overview of the H_{upd}- and CO-based ECSA (GDE) before and after 5k, 15k, and 30k AST.

Sample	$ECSA_{Hupd\text{-}based}$ [m^2/g_{Pt}]				$ECSA_{CO\text{-}based}$ [m^2/g_{Pt}]			
	Before AST	After 5k	After 15k	After 30k	Before AST	After 5k	After 15k	After 30k
PtNi(Mo)/C	31.74	33.10	31.45	27.79	39.37	38.82	35.64	32.45
PtNi(MoRh)/C	36.93	33.09	31.20	29.40	43.85	38.79	36.13	33.78
Hispec3000	56.77	49.24	47.80	44.66	70.13	62.66	58.66	55.28

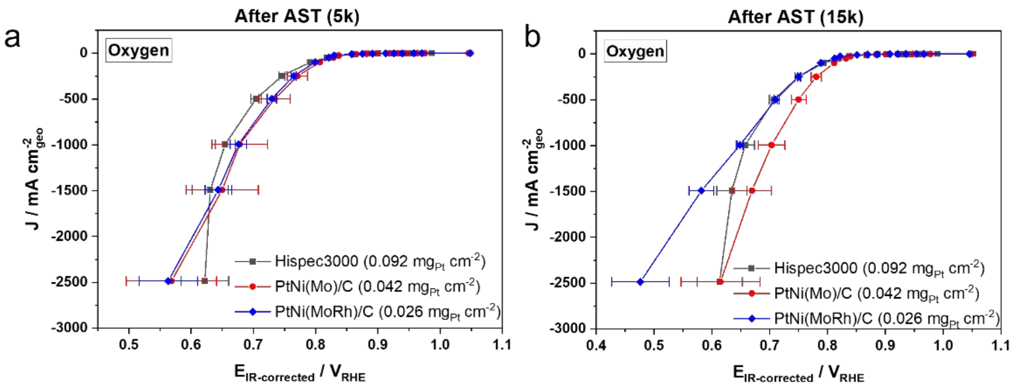

Figure S7: Polarization curves in O_2 of PtNi(Mo)/C (red), PtNi(MoRh)/C (blue), and Hispec3000 (grey) a) after 5,000 and b) after 15,000 cycles.

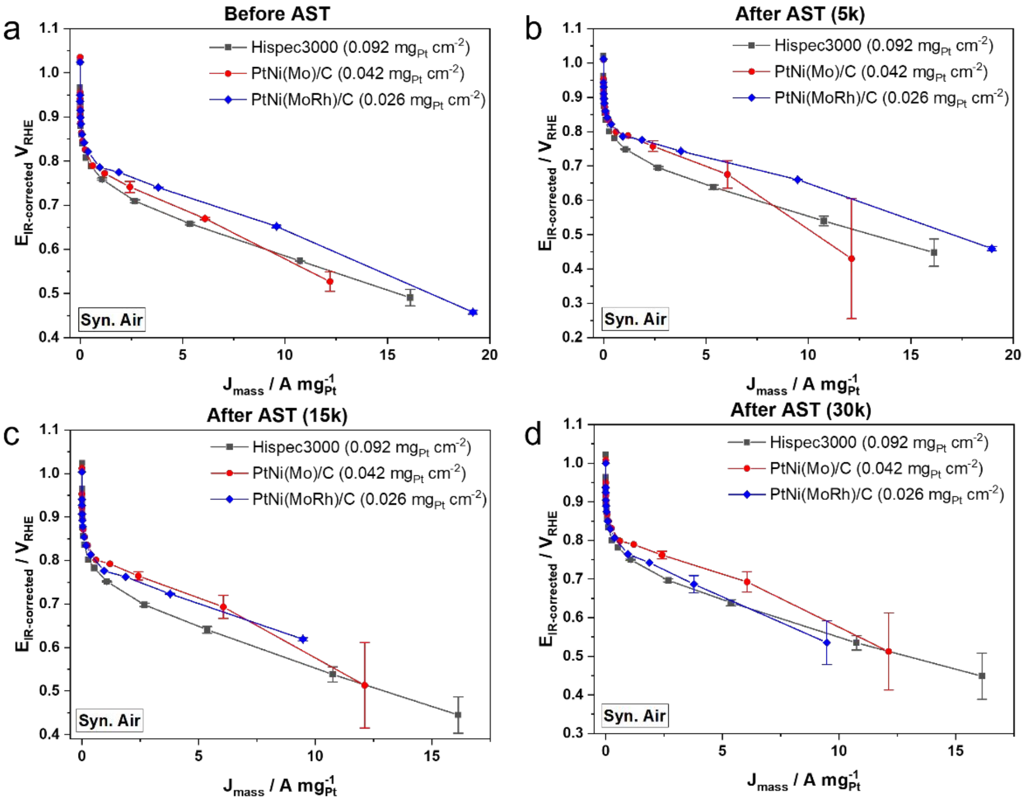

Figure S8: Mass activity curves in synthetic air of PtNi(Mo)/C (red), PtNi(MoRh)/C (blue), and Hispec3000 (grey) at different aging stages: a) before and b) after 5k, c) 15k, and d) 30k AST.

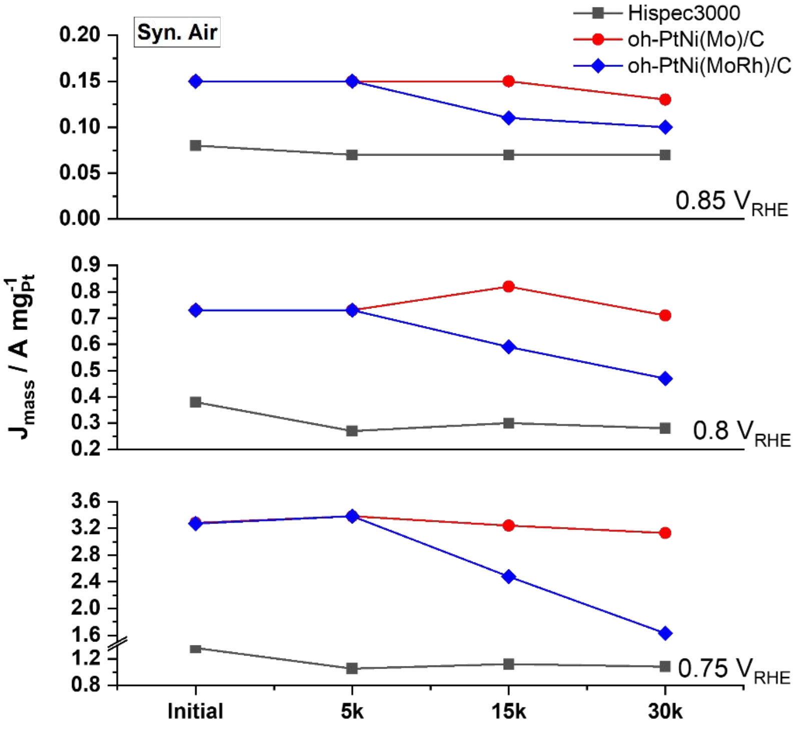

Figure S9: Calculated MAs in Syn. Air before AST and after 5k, 15k and 30k cycles at 0.75, 0.8, and 0.85 V vs. RHE.

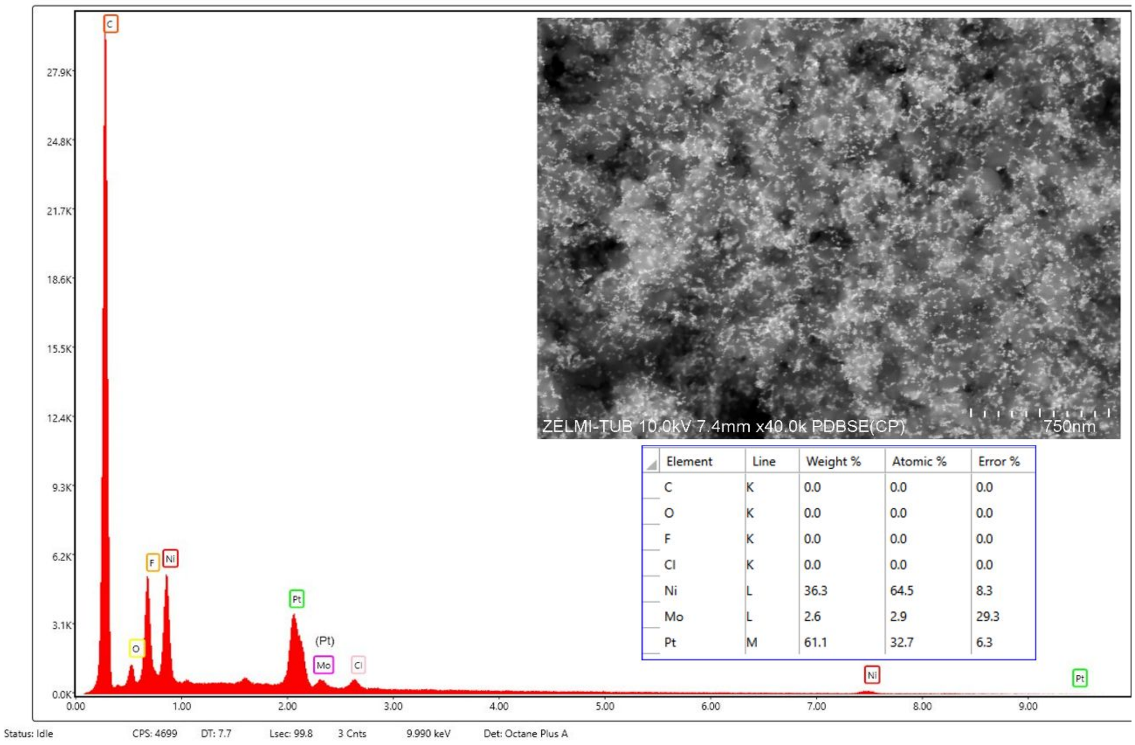

Figure S10: EDX spectrum of PtNi(Mo)/C before AST obtained from the entire image field of the SEM image and the corresponding data of Pt, Ni and Mo in the inset.

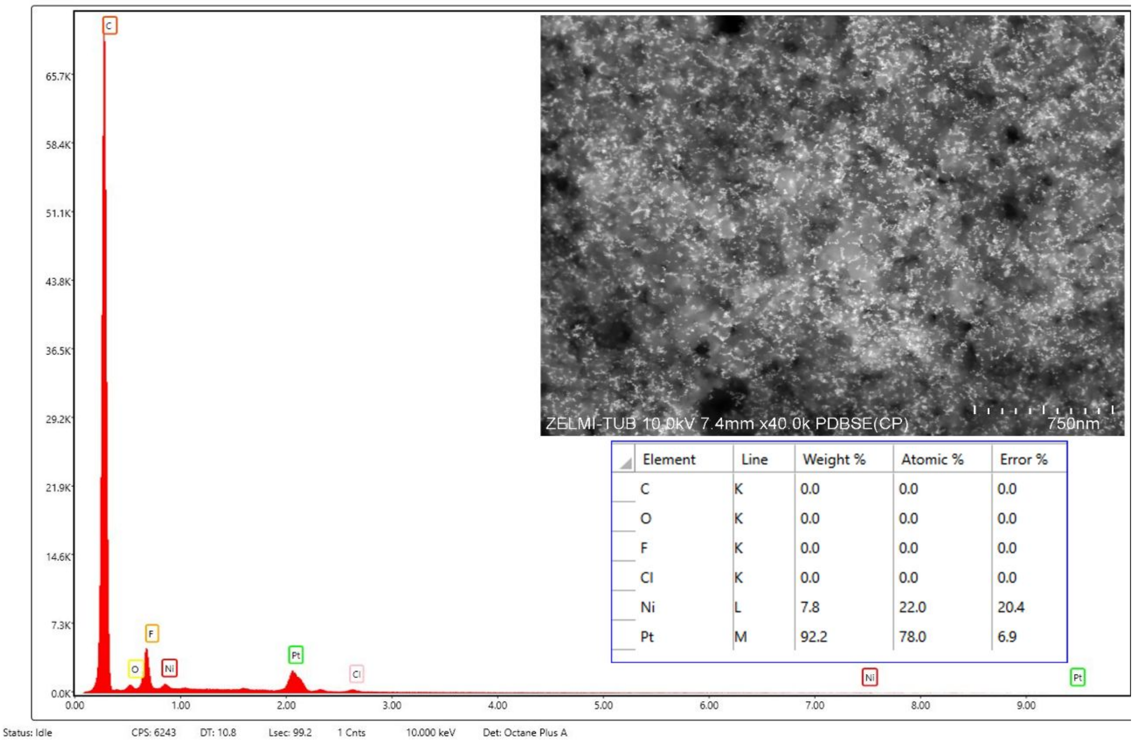

Figure S11: EDX spectrum of PtNi(Mo)/C after AST obtained from the entire image field of the SEM image and the corresponding data of Pt and Ni in the inset.

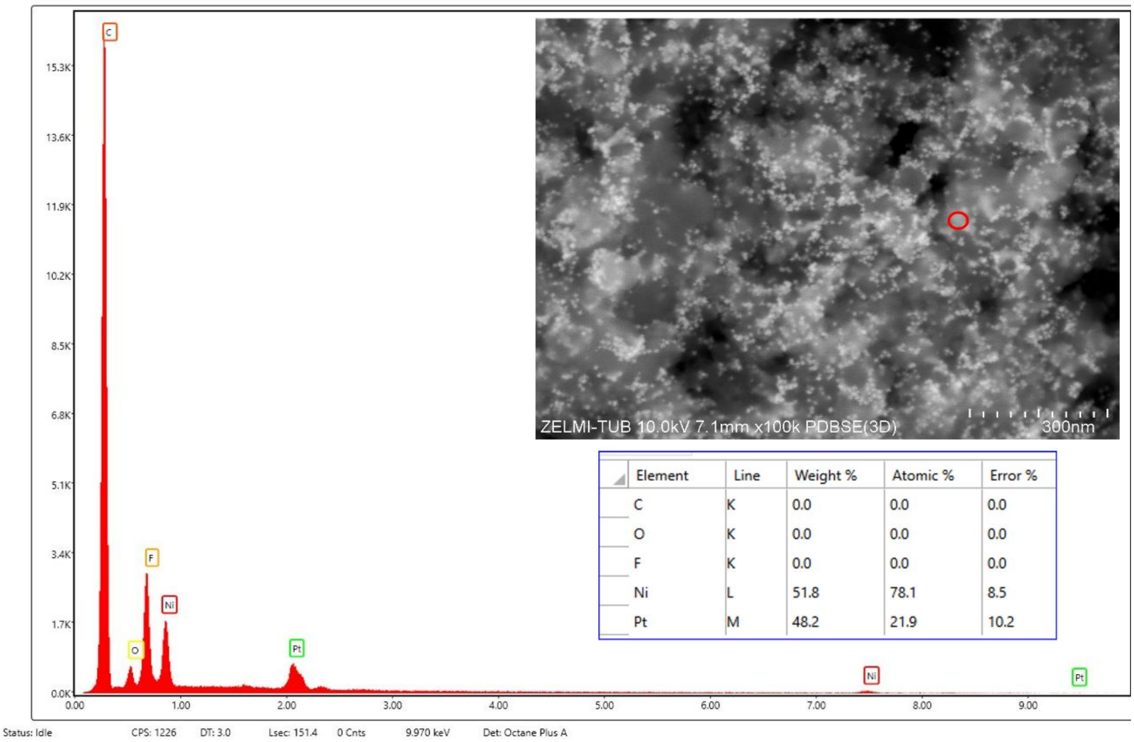

Figure S12: EDX spectrum of PtNi(MoRh)/C before AST obtained with point analysis from the particles (red circle) in the SEM image and the corresponding data of Pt and Ni in the inset.

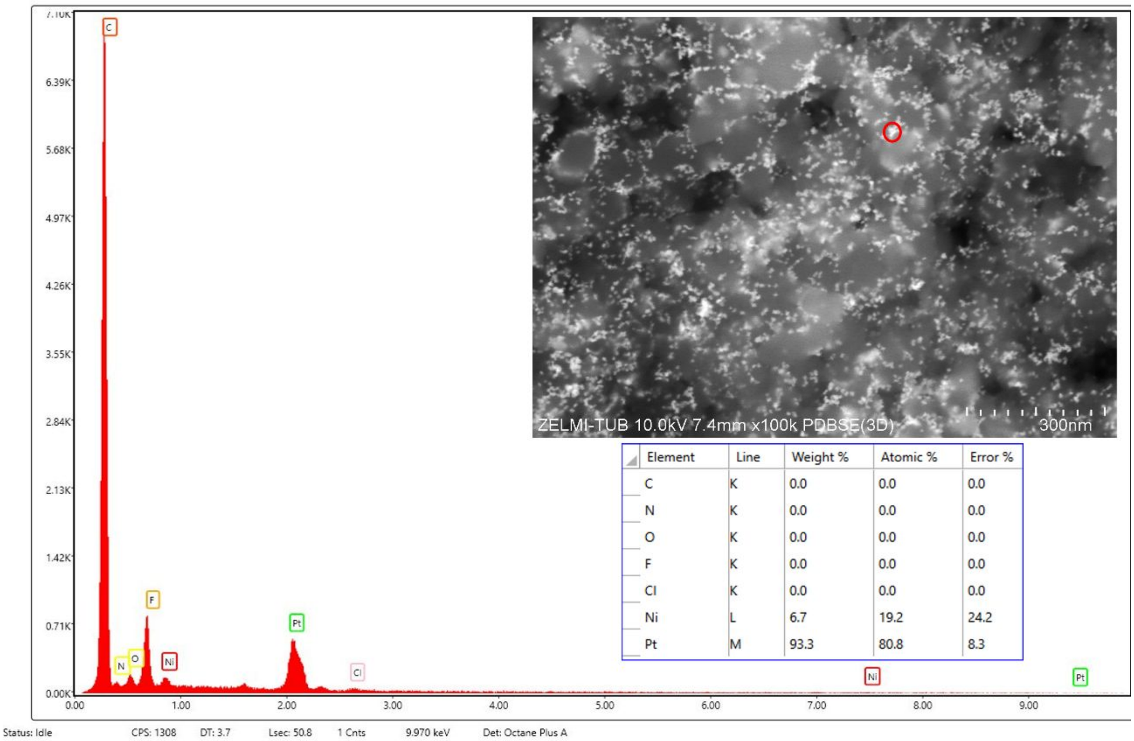

Figure S13: EDX spectrum of PtNi(MoRh)/C after AST obtained with point analysis from the particles (red circle) in the SEM image and the corresponding data of Pt and Ni in the inset.

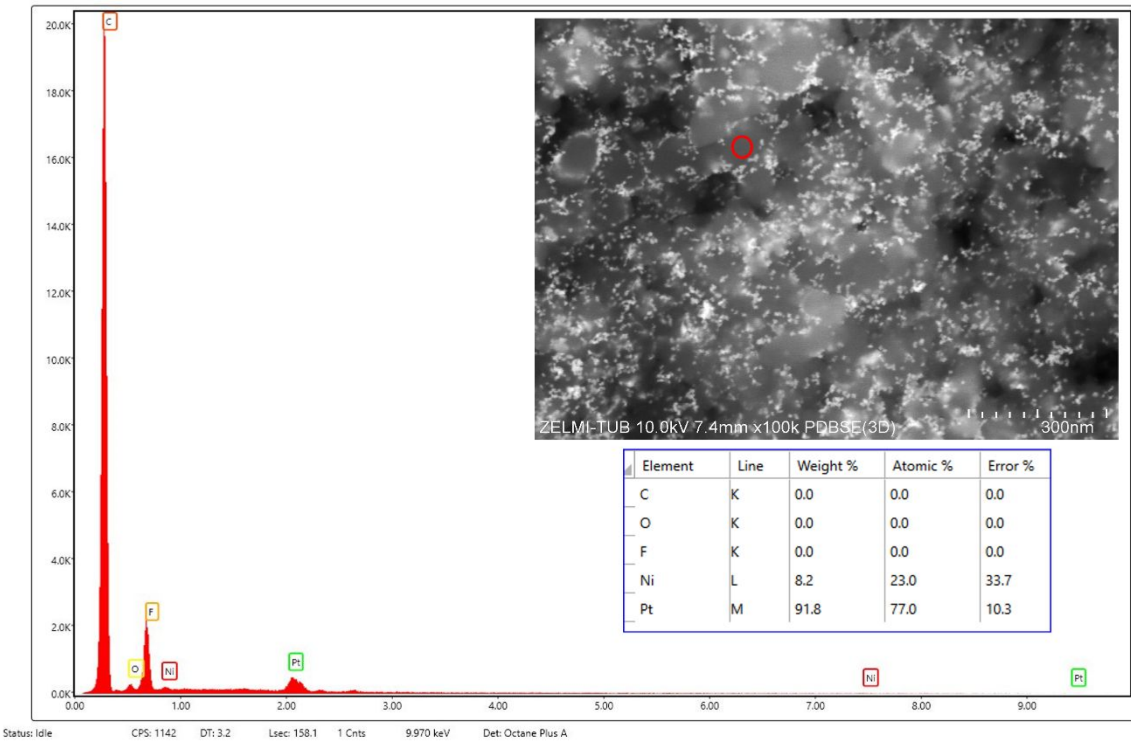

Figure S14: EDX spectrum of PtNi(MoRh)/C after AST obtained with point analysis from the Carbon support (red circle) in the SEM image and the corresponding data of Pt and Ni in the inset.

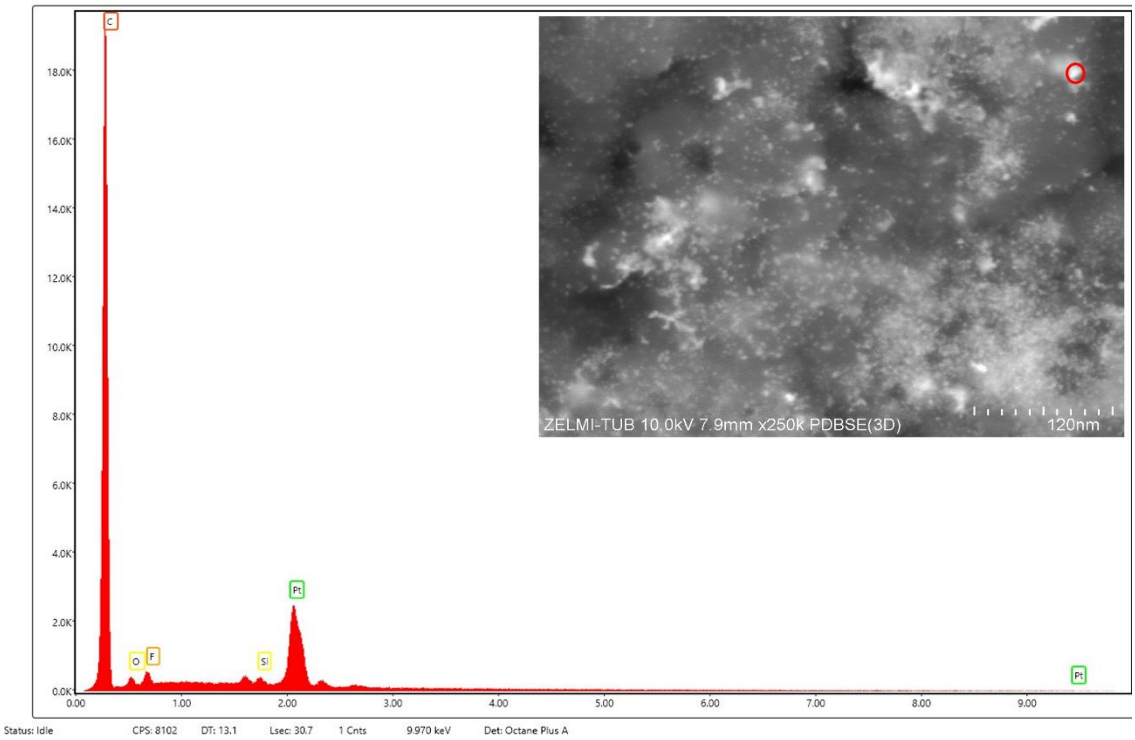

Figure S15: EDX spectrum of Hispec3000 before AST obtained with point analysis from the particles (red circle) in the SEM image.

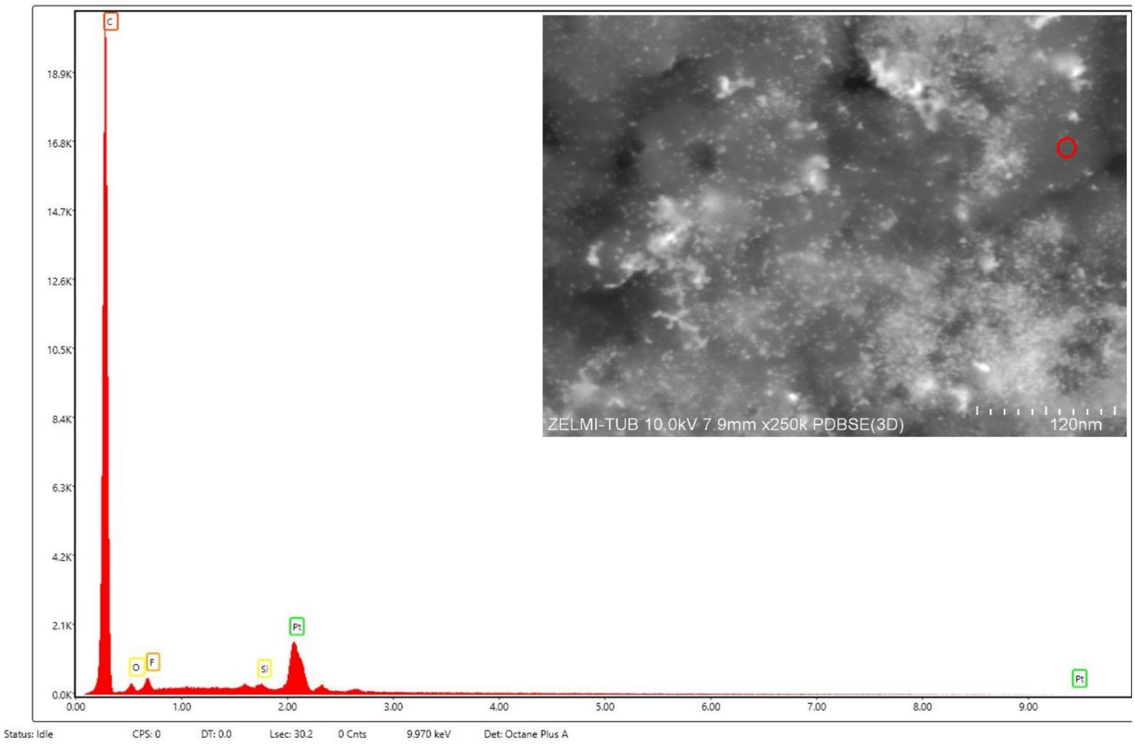

Figure S16: EDX spectrum of Hispec3000 before AST obtained with point analysis from the Carbon support (red circle) in the SEM image.

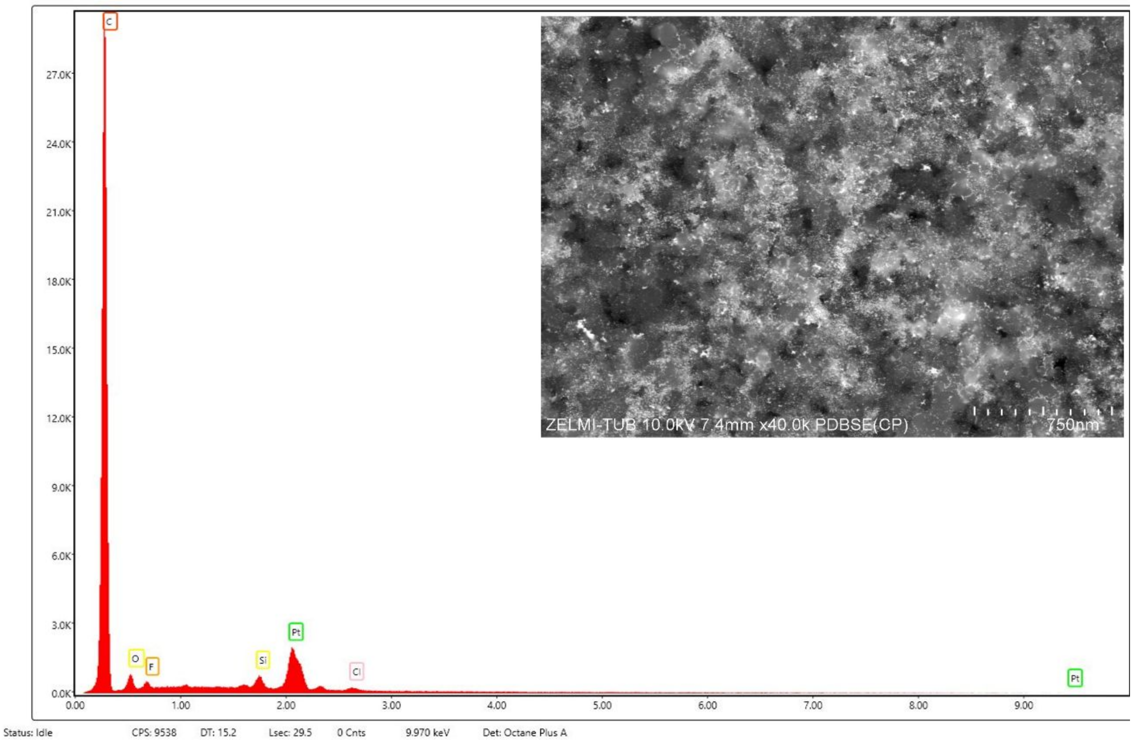

Figure S17: EDX spectrum of Hispec3000 after AST obtained from the entire image field of the SEM image.

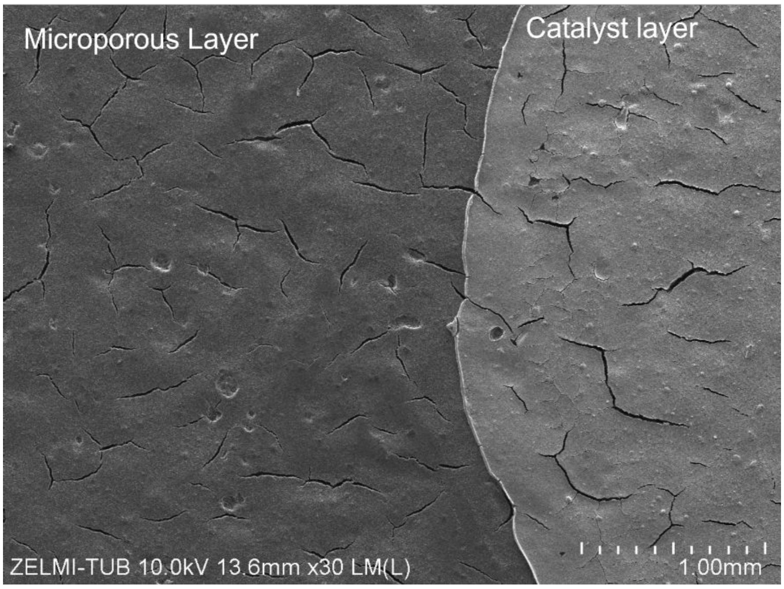

Figure S18: SEM image of a GDE after preparation with microporous layer and catalyst layer.

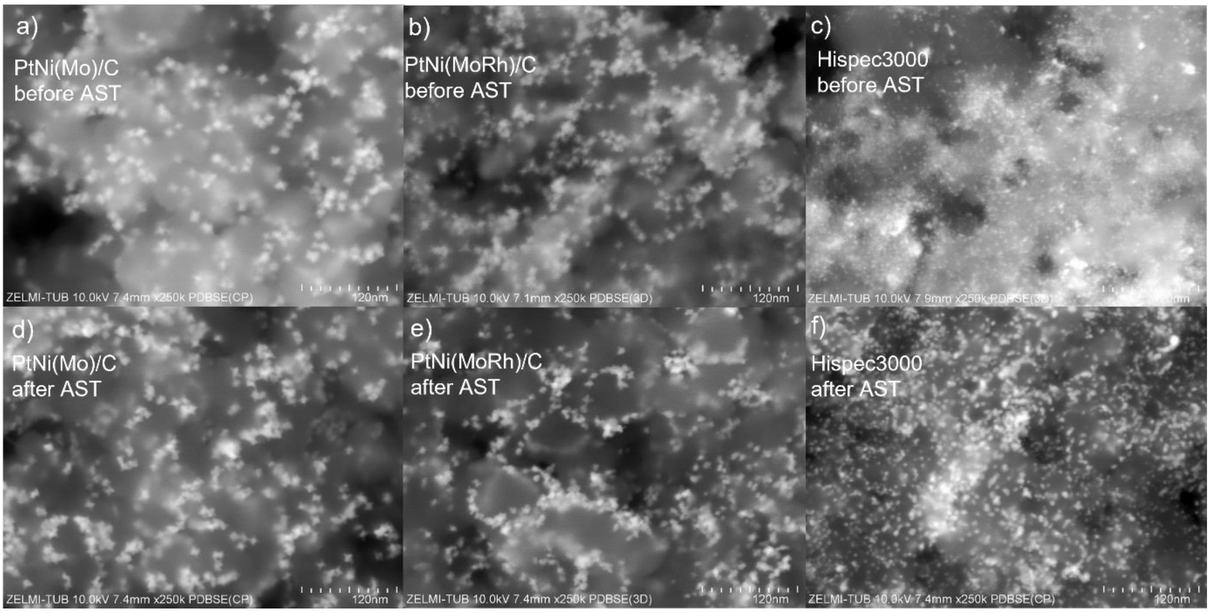

Figure S19: Backscattered electron (BSE) SEM images of PtNi(Mo)/C, PtNi(MoRh)/C, and Hispec3000 a) − c) before AST and d) − f) after AST with a magnification of 250k.

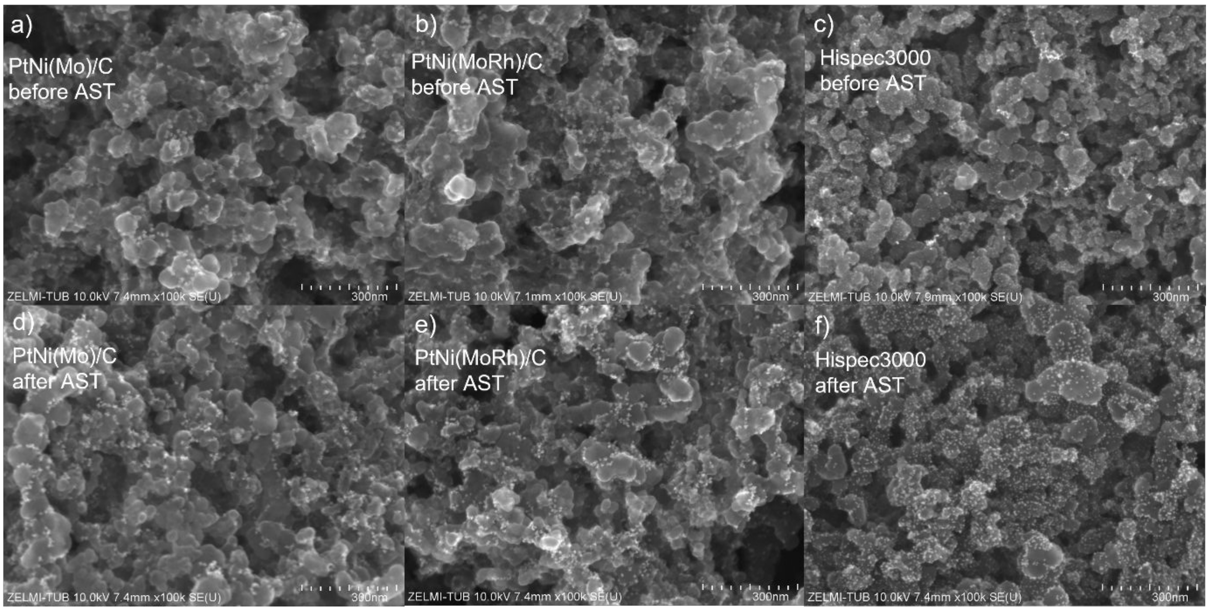

Figure S20: SEM images of PtNi(Mo)/C, PtNi(MoRh)/C, and Hispec3000 a) − c) before AST and d) − f) after AST with a magnification of 100k.

References

1. Rudi, S., Cui, C., Gan, L. & Strasser, P. Comparative Study of the Electrocatalytically Active Surface Areas (ECSAs) of Pt Alloy Nanoparticles Evaluated by Hupd and CO-stripping voltammetry. *Electrocatalysis* **5**, 408–418 (2014).

2. Report, U. D. Fuel cell technologies office multiyear research, development and demonstration plan. *Doe* **2015**, 1–58 (2016).
